# Online Peer Support and Well-being of Mothers and Children: Systematic Scoping Review

**DOI:** 10.2188/jea.JE20200079

**Published:** 2022-02-05

**Authors:** Ai Yamashita, Aya Isumi, Takeo Fujiwara

**Affiliations:** 1 Master of Public Health in Global Health (MPH) Course, Tokyo Medical and Dental University, Tokyo, Japan; 2 Department of Global Health Promotion, Tokyo Medical and Dental University, Tokyo, Japan

**Keywords:** peer support, online, social networking sites, mother

## Abstract

**Background:**

Online peer support groups are common and can be an effective tool for mothers with young children. The purpose of this review is to examine the types of support that online-based peer groups establish, as well as its health effects on mothers and their children.

**Methods:**

Systematic scoping review. Systematic review of existing literature was conducted using PubMed, CINAHL, Medline, Cochrane and Ichushi (Japanese language) database in December 2019.

**Results:**

Based on the inclusion and exclusion criteria, a total of 1,475 articles were extracted by initial search. After the review of titles, abstracts and full texts, a total of 21 articles met the inclusion criteria. The types of support mothers received were mainly informational and emotional support. Mothers also felt a sense of connection and community. Some health effects of online-based peer support group were seen in the area of mothers’ mental well-being. Minimal effects were seen in behavioral modification for child nutrition and breastfeeding.

**Conclusion:**

Due to the limited evidence in interventional studies, the effects of online-based peer support groups were inconclusive. Further studies with rigorous research designs would be helpful in future research.

## BACKGROUND

Isolation of mothers, especially those with young children, in communities/societies has been a frequently discussed topic.^1^^,^^2^ Shifts in family/community structure and change in workstyle for women contribute to social isolation among new mothers.^3^^,^^4^ The geographic loss of a community network means fewer formal and informal safety nets for mothers.^2^^,^^3^^,^^5^ Birthing and parenting can be physically and mentally demanding for mothers. Although the duration of postpartum hospitalization and the quality of postpartum care varies from country to country, the mothers with newborns are discharged soon after the delivery and are expected to know how to look after their infants. There used to be female family members and relatives who could share the wealth of wisdom for childbearing and childrearing at home or in the neighborhood; however, due to change in social structure, such as increases in the number of nuclear families, single-parent households, and pregnancies at advanced maternal age, it is more and more difficult for modern mothers to obtain readily available information and advice from their strong ties.^6^^–^^8^

With this trend, internet appears to be filling the gap of mothers’ needs for information and advice. Internet technology has spread dramatically over the past two decades and many parents utilize the Internet to seek information and support regarding health and parenting.^9^^,^^10^ Numerous web-based mothers’ communities have been established, formally and informally, and mothers use those sites for various reasons, including anonymity and immediate affirmation and support.^9^^,^^11^ These online communities take various forms, such as bulletin boards, email threads, blogs and Social Networking Site (SNS) communities. SNS communities have grown significantly and many participate in one or more types of SNS.^12^ In addition to the Internet, SNS has also become a critical source of information and support for parents.^9^^,^^11^ Thus researchers have recently revealed how mothers of young children use the Internet and SNS, yet the effectiveness of online peer support groups on their well-being has not been well-examined.

Furthermore, it is important for health professionals including public health nurses to understand the effectiveness of online peer support groups because professional-led interventions were conducted online, sometimes in conjunction with online peer support groups.^13^^,^^14^ Also, online peer support effects of general mothers are even less clear since many studies in this field were based on underline conditions and diseases.^4^^,^^15^^,^^16^ By performing a review of currently available publications, the following questions are explored:

1. What kind of peer support are mothers providing and receiving from online parenting communities?
2. Are there any health effects to mothers and their children in relation to the use of online parenting communities?

## MATERIALS AND METHODS

### Inclusion and exclusion criteria

With this scoping review, articles were searched for online peer support for infant and young children. Peer-reviewed journals in English and Japanese were included in the review. The inclusion criteria were as follows:

1. The study focused on online peer support group.
2. Online-based peer support was a part of interventions and/or focus of the study.
3. The target population included mothers with preschool children or younger.
4. The article assessed/examined the effect of online-based peer support group for mothers.

Exclusion criteria were as follows:

1. The target online-based peer support groups were based on clinical conditions, diseases or prevention of specific clinical/health conditions.
2. The target online-based peer support groups have a major healthcare professional component (eg professional-led interventions)

**Figure 1.**
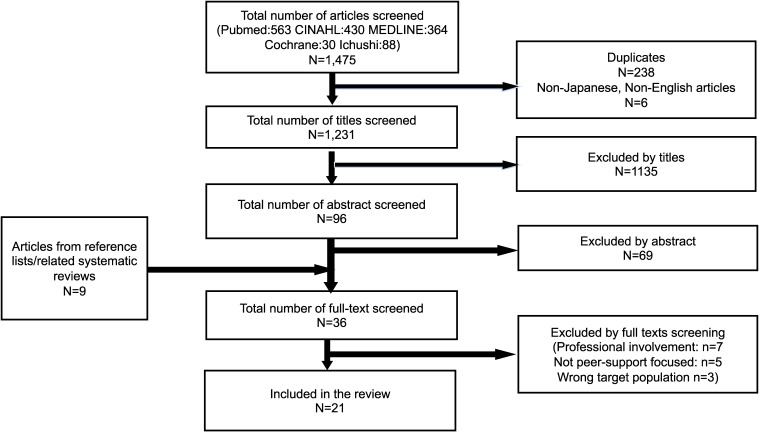
Publication Screening Process Diagram

Literature selection processes are shown in Figure 1.

### Search strategy

The literature search was conducted in December 2019. Pubmed, Medline, CINAHL, Cochrane and Ichushi (Japanese language) database were screened. Search terms used for the literature search is listed in Table 1. No limitation on years were applied. Some common clinical conditions related to online peer support, such as developmental disabilities, rare diseases, substance abuse, and neonatal intensive care (NICU) were applied as exclusion criteria. Elderly population was also applied as an exclusion term, since the target population was mothers of young children. With the database screening, a total of 1,475 articles were extracted for the review.

**Table 1.**
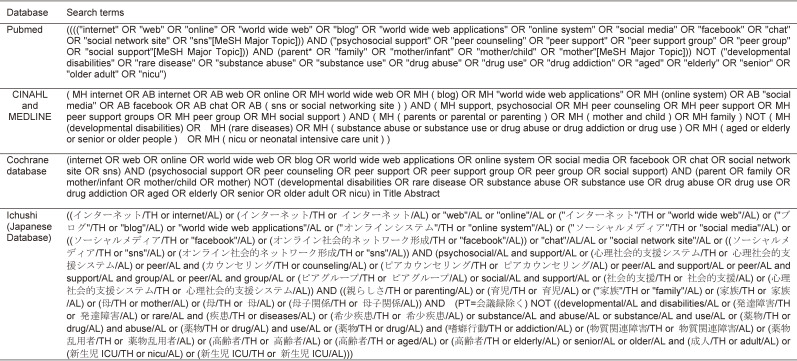
Search terms used in literature database

Among the selected articles, 238 articles were excluded due to duplication. Titles were screened for appropriateness for the review based on inclusion/exclusion criteria. Articles were excluded if the title included specific diseases names (ie, diabetes, autism), target population other than mothers with young children (ie, fathers, adolescents), terms that indicate specific treatment/intervention aims (ie, abuse, grief), or terms that indicate types of research or intervention other than online peer support for parents (ie, systematic review, intervention for children). Then abstracts were examined based on inclusion/exclusion criteria (*n* = 96). The remaining 27 articles plus nine articles added from reference lists and other related systematic reviews were examined in full text based on the same criteria. After completion of the review with two researchers, a total of 21 articles were selected for this review (Figure 1).

## RESULTS

With the selected articles, most often seen were qualitative studies with web posts analysis^2^^,^^17^^,^^18^ and interviews.^19^^–^^27^ Eight articles were quantitative studies^8^^,^^28^^–^^34^ and one utilized mixed methods.^35^ One-third of the selected articles were from Australia.^17^^,^^19^^,^^20^^,^^24^^,^^27^^–^^29^ There were four publications each from Canada^18^^,^^22^^,^^23^^,^^35^ and the United States,^2^^,^^30^^,^^32^^,^^34^ three from the United Kingdom,^21^^,^^25^^,^^26^ two from Japan,^8^^,^^31^ and one from China.^33^ The years of the publications ranged from 1998 to 2019. Four interventional online-based peer group platforms were created by researchers.^17^^,^^28^^,^^29^^,^^35^ Description of the studies and outcomes are listed in Table 2.

**Table 2.** Online-based peer support group for mothers of young children

	Author year country	Research design, Intervention or support group	Methodological approach sample size	Outcomes	Significant (+) or non-significant (−) results	Reference Number
**Online group created by researchers**						

1	Downing, KL (2017) Australia	• Retrospective cohort study • Facebook peer support group	• Participants: first-time mothers (with children between 18 to 36 months) • Comparison of mothers who joined FB group (*n* = 102) and non-FB group (*n* = 53)	• BMI (kg/m^2^)	−	28
				• Waist circumference	−	
				• Fruit intake (difference *P* = 0.047 122.6 g/day in control vs 152.7 g/day in intervention group)	+	
				• Vegetable intake	−	
				• Water intake	−	
				• Non-core drink intake	−	
				• Non-core sweet snack intake	−	
				• Non-core savory snack intake	−	
				• Energy intake	−	
				• Television viewing	−	
				• LMVPA (light-, moderate-, and vigorous-intensity physical activity.)	−	

2	Giglia, R (2015) Australia	• Nested randomized study within longitudinal cohort study • Up to 26-week follow ups post-birth	• Participants: Mothers of newborns (*n* = 514) • Intervention group had access to discussion forum, email to other members • Control group (*n* = 207) and intervention group (*n* = 207) with pre and post-tests	Exclusive breastfeeding at:		29
				• 4 weeks after delivery	−	
				• 10 weeks after delivery	−	
				• 16 weeks after delivery	−	
				• 26 weeks after delivery (difference *P* = 0.01: 5.6% of intervention group vs 0.6% of control group)	+	

3	Hudson, DB (2009) Australia	• Qualitative phenomenological study • Original web-based discussion forum	• Participants: adolescent single, low-income mothers with 1-week old infants (*n* = 20) • Content analysis of web posts	• support of family and friends (physical support and advice)	+	17
				• Criticism	−	
				• peer support (emotional and instrumental support)	+	
				• system support (receive answers to technical questions)	+	

4	Dunham, PJ (1998) Canada	• Mixed study (pre- and post-test and qualitative ethnographical study). • Original online bulletin boards	• Participants: single, adolescent mothers with newborns (*n* = 42) • Comments analysis and questionnaires	Parental stress		35
				• significant relationship was seen between active online participation and decreased PSI (χ^2^ = 6.05, *P* < 0.04)	+	
				Qualitative:		
				• Majority of postings were positive and supportive (97.9%) and positive messages were Emotional support (56%), Informational support (37%), and Instrumental support (3%)	+	

**Existing online communities**						

5	Drentea, P (2005) USA	• Qualitative phenomenological study (some descriptive statistics data) • Parenting website (bulletin boards).	• Participants: web group mothers of infants up to 6 months (*n* = 180) • content analysis of web posts	• Increases social capital during the time when women are isolated as new mothers.	+	2
				• Social capital operates through emotional support, information-giving, and community protection to aid mothers of infants.	+	

6	Holtz, B (2015) USA	• Cross sectional study with online questionnaires • FB discussion group	• Participants: pregnant women or mothers with children under five years old (*n* = 647) • Factor analysis with a latent variable framework	Online engagement was correlated with:		30
				• Feeling of social support (*P* < 0.001)	+	
				• Empowerment (*P* = 0.001) ^*^RMSEA = 0.035, CFI = 0.980, TLI = 0.978	+	

7	Miyata, K (2002) Japan	• Quantitative longitudinal panel design study. • Online forums (Nifty)	• Participants: Mothers of preschool children exchanging childcare information online (*n* = 331) • Two questionnaires in three-month interval for comparison	Structural equation model (SEM) showed correlation between internet support and:		31
				• Depression (factor loading: −0.17)	+	
				• Self-esteem (factor loading: 0.21) ^*^mothers who receive more internet support demonstrate more well-being than those who receive less internet support.	+	

8	McDaniel, BT (2012) USA	• Cross sectional study with online survey with purposive sampling. • Mothers using SNS and blog	• Participants: first-time mothers with children under 18-month old (*n* = 157) • Structural equation Model for analysis	blogging indirectly (through connection with family/friends and social support) influence:		32
				• marital satisfaction (factor loading: 0.26)	+	
				• marital conflict (factor loading: −0.33)	+	
				• parental stress (factor loading: −0.19)	+	
				• maternal depression through parental stress (factor loading: −0.19)	+	

9	Hall, W (2008) Canada	• Phenomenological study • Online forum	• Pregnant women and mothers of newborns (up to 11-month-old) (*n* = 40) • Content analysis of online posts	• request and provide emotional support	+	18
				• share information and facilitate learning	+	
				• provide validation for the ‘normalcy’ of other women’s mothering experiences.	+	

10	Bridges, N (2016) Australia	• Ethnographic study • breastfeeding online support group	• Mothers breastfeeding and participating target online support group (*n* = 23) • Thematic analysis of interview and focus group data	Overreaching theme: “community”	+	19

11	Nolan, S (2015) Australia	• Phenomenological study with narrative inquiry • SNS users	• Samples are adolescent mothers (16–19 years old) with one child (3–17-month-old) (*n* = 7) • Interview (Narrative inquiries) • Constant comparison analysis method	Themes emerged:		20
				• Social connectedness	+	
				• Increased parenting confidence	+	
				• Reduced parental stress	+	
				• Enhanced self-disclosure (anonymity)	+	
				• Access to information	+	

12	O’Connor, H (2004) UK	• Phenomenological study • Online community	• Online group interviews with new mothers (*n* = 16) • Children’s ages are unknown • Inductive analysis of interview contents	• Shared experience	+	21
				• Virtual social support	+	
				• Empowerment	+	
				• Non-judgmental safe place (anonymity)	+	

13	Valtchanov, BL (2014) Canada	• Grounded theory study • Online community	• Samples are mothers of infants (*n* = 22). • Data from individual interview • Charmaz’s (2008) constructivist grounded theory approach	Technologically mediated motherhood and creating communities online:		22
				• “Virtual neighborhood”: Changing communities	+	
				• “Stuck at home”: Accessible online community	+	
				• “Moms support network”: Supportive online community	+	

**No specific target online communities**						

14	An, Z (2016) China	• Cross sectional study • No specific targeting online group	• Purposive sampling of first-time mother born in 1980s (*n* = 366). • Children are younger than three years old • Quantitative structural equation modeling (SEM).	SEM (χ^2^_(95)_ = 159.20, *P* < 0.001, RMSEA = 0.04, 90% CI[0.03, 0.05], CFI = 0.97, GFI = 0.95, IFI = 0.97 with Δχ^2^_(13)_ = 21.81):		33
				• Online support predicts reduced perceived stress (β = 0.09, ns)	−	

15	Mandai, M (2018) Japan	• Cohort cross sectional study • No specific targeting online group	• Mothers with children under 3 years old (*n* = 523) • Questionnaire analysis	Loneliness relates to:		8
				• Smaller SNS network (ρ = −0.33, *P* < 0.001)	+	

16	Jang, Y (2014) USA	• Cross-sectional study (online survey) • No specific targeting online group selected	• Subsample of national LISTSERV • Mothers who use SNS and their oldest children is 18 years old or younger (*n* = 665). Among them, mothers of preschool children are 283. • Path analysis	Path analysis model for mothers of preschool children (χ2 (24) = 32.39, *P* = 0.12, CFI = 0.95, RMSEA = 0.04):		34
				• Frequency of using SNS (B = 0.14, *P* < 0.05) and number of SNS activities (B = 0.11, *P* < 0.001) are positively associated with bonding social capital	+	
				• Frequency of using SNS (B = 0.24, *P* < 0.01) and number of SNS activities (B = 0.20, *P* < 0.001) are positively associated with bridging social capital.	+	

17	Price, SL (2018) Canada	• Qualitative phenomenological study • No specific targeting online group	• Convenient sampling of first-time mothers (*n* = 18) with infants under 1 year old. • Data collected with focus group and e-interview.	• Maternal knowing (normalization and intuition)	+	23
				• Emergence of personal knowing and social networks	+	
				• ^*^online activities complement face-to-face interaction	+	
				• Importance of nonhierarchical relationship	+	

18	Johnson, SA (2015) Australia	• Qualitative phenomenological study • No specific targeting online group selected	• Samples consists of first-time mothers (*n* = 12) • Data was obtained from prenatal (32–38 weeks) and postnatal interviews (when infants are 3 to 7 months).	• Value experiential knowledge and practical advice.	+	24
				• Safe space for women for problem solving (anonymity and filtering of knowledge)	+	
				• Gaining by passive participation (surreptitious support)	+	
				• Assembling community	+	

19	Gibson L (2013) UK	• Qualitative ethnographic study. • No specific targeting online group selected	• Mothers who delivered within 12 months (*n* = 6). Samples were collected from face-to-face parenting group. • Children <1 year-old • Data was collected via observation and interview	• Improving confidence as a mother: seeking advice.	+	25
				• Breaking into the new community; technology for parenting.	+	

20	Regan, S (2019) UK	• Qualitative study with semi-structured interviews. • Facebook or other online interactive breastfeeding support forum users.	• ≥18 years old mothers who currently breastfeeding or breastfeeding in the past (*n* = 14). • Children up to 3 years old • Data was collected via semi-structured individual interviews • Thematic analysis of interviewed contents	• Reassurance and normalizing	+	26
				• Someone who has been through it	+	
				• Circle peer support	+	
				• Judgement	−	
				• Polarized debate	−	
				• Lack of regulation	−	

21	Lupton (2016) Australia	• Qualitative Study • Focus group interviews	• 27 mothers with young children under 3 and 9 women with first pregnancy. • Inductive thematic analysis of discussion contents	• Intimate information exchange	+	27
				• Reassuring information exchange	+	

CFI, Comparative Fit Index; RMSEA, Root Mean Square Error of Approximation; SNS, Social Networking Site.

### Study interests

There were several studies focused on first-time mothers.^20^^,^^21^^,^^23^^,^^24^^,^^28^^,^^32^^,^^33^ Some targeted vulnerable populations such as adolescent and/or single mothers.^17^^,^^20^^,^^35^ Among the intervention-based studies, outcome measures extended to various areas such as nutrition of children,^28^ initiation and duration of breastfeeding^22^ and parental stress level^29^ as well as social support.^17^^,^^35^ For non-interventional quantitative studies, research data was mainly retrieved from cross-sectional questionnaires/surveys.^8^^,^^30^^–^^33^ For qualitative studies, interviews, questionnaires and focus group meetings were the main data sources. Three studies retrieved data from online contents.^2^^,^^17^^,^^18^ The majority of studies had outcomes measures with mental health related topics including empowerment,^21^^,^^30^ depression,^31^^,^^32^ self-esteem,^31^ self-confidence,^20^^,^^25^ parental stress level,^20^^,^^32^^,^^33^^,^^35^ social support,^2^^,^^18^^,^^21^^,^^30^^–^^33^ and loneliness.^8^

### Outcomes and emerging themes

#### Mothers’ mental well-being: feeling of support from online groups

The majority of articles discussed direct or indirect effects of social support (informational, emotional and instrumental).^2^^,^^17^^,^^18^^,^^21^^,^^30^^–^^33^^,^^35^ Social capital was examined by Jang^34^ by conducting path analysis among mothers of pre-school children in the United States. This study found that frequency of SNS use had a positive association with bonding social capital and bridging social capital. Drentea et al^2^ analyzed contents of mothers’ online bulletin boards threads and concluded that online community increased social capital by exchanging information and emotional support as well as community protection. Others suggested that mothers felt connectedness.^20^ Yet, some studies also acknowledged negative support such as criticism, disagreement, polarized debate, and judgement, were seen in online communities.^2^^,^^17^^,^^27^^,^^35^

#### Mothers’ mental well-being: parental stress level and maternal depression

There were three studies that examined relationships between online peer support and parental stress.^32^^,^^33^^,^^35^ Nolan^20^ conducted a qualitative research targeting adolescent mothers and found parental stress reduction with utilization of web discussion. Those mothers felt the effect of stress reduction by sharing problems and receiving positive feedback and empathetic responses. With the study conducted by Dunham,^35^ Parenting Stress Index (PSI) was measured before and after the participation of web moderated social network platform for 6 months. The result showed that active participants were more likely to have decreased stress level post-intervention. Another study assessed parenting stress in relation to participation in blogging and online social networking among first time mothers.^32^ There was no direct significant association between parenting stress and blogging/social networking. However, the author pointed out that blogging frequency predicted feelings of connectedness and connectedness predicted the social support when the variables were fitted to structural equation model (SEM). Parental stress had an intervening effect between social support and maternal depression.

Miyata^31^ explored depression and found that “non-posting” mothers in online peer groups initially had higher depression score, but the score lowered after three months. Meantime, depression score for “posting” group remained relatively same. When fitted into SEM, it was suggested that internet support may have indirectly reduced depression among online group users. A study done with Chinese mothers; however, did not support a hypothesis of negative relationship between mothers’ online activities and perceived stress level.^33^

#### Mothers’ mental well-being: feeling of empowerment, self-esteem, and confidence

Some also suggested that mothers not only accessed online communities to obtain knowledge, but also to seek validation for normality.^18^^,^^23^^,^^26^ Empowerment was another topic examined in relation to mothers’ online activities. One study showed online group participation was correlated with feelings of empowerment among mothers.^30^ O’Connor et al^21^ also pointed out that virtual community provided an additional source of advice and increased mothers’ sense of empowerment. In this study, association between online group engagement and feeling of empowerment was positive.

As for self-esteem, Miyata suggested an indirect effect on self-esteem with internet community participation and this was also true with the participants who did not actively participate.^31^ Some qualitative studies indicated that online community helped to increase mother’s confidence as a parent.^20^^,^^25^

#### Mothers’ mental well-being: loneliness

Mandai et al^8^ conducted cohort cross-sectional study of Japanese mothers and found that higher loneliness was significantly related to smaller SNS (social network sites) network as well as real social network.

#### Behavioral change facilitation with online-based peer group

Some studies assessed behavioral outcomes with online-based peer group interventions. Those included child nutritional intake^28^ and breastfeeding.^29^ Family/social network related outcomes were also measured and those included marital satisfaction/conflict^32^ and family support.^33^

#### Behavioral change facilitation: nutritional intake of children

Downing et al^28^ examined Facebook peer group support effects after a series of nutrition classes (face-to-face). In this study, primary outcomes were children’s physical measurements, physical activities and various food intakes. The amount of fruit intake was the only significant difference noted between mothers with online peer support and non-peer group support. Fruit intake was increased among children with mothers who joined online peer support group.

#### Behavioral change facilitation: breastfeeding

A nested interventional study was conducted to assess exclusive breastfeeding.^29^ Intervention group had access to other members, and they had significantly higher exclusive breastfeeding rate at 26 weeks after delivery though no significance was noted in any other weeks.

## DISCUSSION

Online peer support in form of informational, emotional supports were consistently present in the reviewed studies. Online peer support communities seem to influence mothers’ mental well-being directly and indirectly. Yet, interventions related to behavioral change focusing on feeding did not seem to have significant effects. Price et al^23^ concluded that online peer support would complement face-to-face interaction, but not as a substitution. Yet, others valued the nature of online anonymity and this particular environment provided safe spaces for mothers and facilitated disclosure of their true feelings and concerns.^20^^,^^21^^,^^24^^,^^27^

One of the benefits of online support is its accessibility. Mothers can have a better sense of control and peace of mind by obtaining information, advice and reassurance in timely manner. There are “lurkers” who do not actively participate in the online communities but follow and obtain information as needed by browsing online sites. Miyata^31^ and Johnson^24^ suggested that those passive participants could also receive some benefits from online communities.

Accuracy of online information often becomes a focus of discussion, but the trend of online information usage among mothers may be slightly different from the information provided by healthcare professionals. Mothers use online information for validation or as an additional information source. Johnson^24^ suggested that mothers did not take all the information they received online. They rather filtered the information according to their needs. Price et al^23^ also suggested mothers gather information they need and follow their intuition for their decision making. Mothers may be asking questions online not because they want to get the right answers, but to gather heterogeneous opinions. It can also play a role of safety nets for mothers who have common questions which can be answered by other mothers who went through the same situation. However, online information can be misleading, unhelpful, or even wrong in some cases,^9^^,^^10^ and mothers’ information literacy skills may play a critical role for online information to be effective. Also, it is reported that mothers’ problematic use of the Internet or/and SNS (ie, addiction) has a detrimental impact on their interpersonal relationships and emotional stability, which could possibly lead to child maltreatment.^36^ In addition to understanding these negative aspects online peer support may have, it is especially important for healthcare professionals to consider when promoting maternal health through peer-support channels.

### Limitations

There were limited numbers of interventional studies evaluating effectiveness of online peer support. Because of this, the review took the scoping review to explore what was already known in this field of research, and comparison by country or year the research was conducted was not made. Given that culture and social systems can be different depending on countries and the environment of parents, especially their usage of the Internet and SNS, can change over time, future studies should consider them in examining effectiveness of online peer support groups. Due to the nature of the review, no critical appraisal or bias assessment was conducted.

### Conclusion

Effects of online-based peer support groups for mothers were explored. The review suggested some positive effects on maternal mental well-being, but the evidence was very limited to properly evaluate effectiveness of online peer group among the mothers of young children. When healthcare professionals consider health promotion of mothers through an online-based peer support group approach, the unique needs of mothers-to-mother support should be considered to maximize the support efforts.

Internet continues to be one of the major information sources for parenting mothers. Online peer support can be an easy and convenient way to increase maternal mental well-being. It gives mothers a unique opportunity to connect with others and exchange opinions. It can be particularly helpful in the circumstance where in-person social networks are limited, including the situation of COVID-19 pandemic. More structured interventional study designs to evaluate the effectiveness of online peer support are needed.
